# Supplementary material for: Electroacupuncture improves swallowing function in a post-stroke dysphagia mouse model by activating the motor cortex inputs to the nucleus tractus solitarii through the parabrachial nuclei
Source: Nat Commun. 2023 Feb 13;14:810. doi: 10.1038/s41467-023-36448-6 (PMC9925820; doi:10.1038/s41467-023-36448-6)
Supplement: Supplementary file 1 — Supplementary Information [file 41467_2023_36448_MOESM1_ESM.pdf]

Supplementary figures

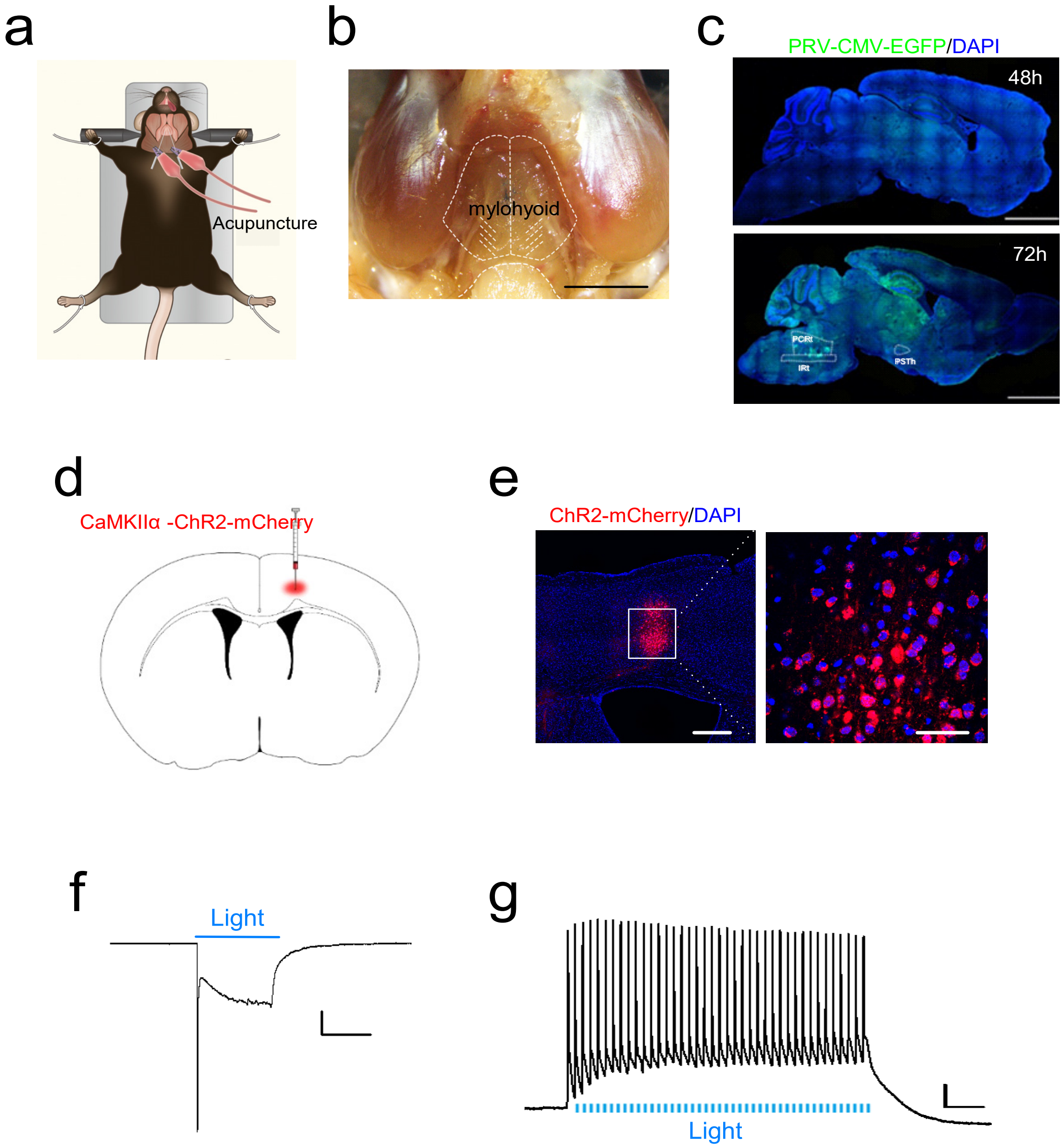

Sup Fig. 1. Identification of synaptic connections between the M1 and the mylohyoid.

(a) The position of the needle for acupuncture at CV23. **(b)** Confirmation of the location of dye injection (Fast Green, FCF). Scale bar, 4 mm. **(c)** The distribution of trans-synaptically labeled neurons in the M1 at 48 h and 72 h after PRV injection into the mylohyoid. Scale bar, 2500 μm. **(d)** The experimental scheme for AAV2/9-CaMKIIα-ChR2-mCherry injection. **(e)** Representative images showing neurons expressing AAV2/9-ChR2-mCherry in the M1. Scale bar, 500 μm (left) and 50 μm (right). **(f)**

Representative traces obtained by *in vitro* slice electrophysiological recording showing that membrane potential was depolarized by optogenetic activation of ChR2-expressing excitatory neurons in the M1 by a blue laser (473 nm). The blue line represents the light delivery for 300 ms duration. Scale bar, 200 ms and 1000 pA. **(g)** The action potential firing in a neuron in response to blue light stimulation at 50 Hz. Scale bar, 200 ms and 20 mV.

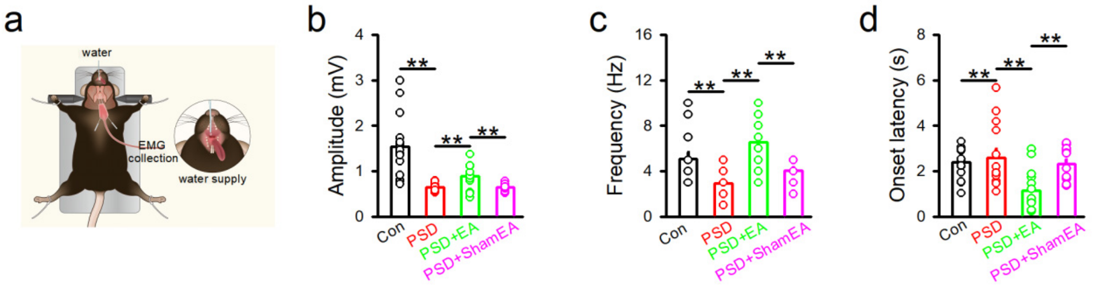

Sup Fig.2. Characterization of water-induced EMG responses showing the effect of EA treatment on swallowing function.

(a) Schematic diagram showing the placement of the electrode and water delivery during EMG recording. **(b)** The average amplitude of EMG responses in the control (Con), PSD, PSD and EA treatment (PSD+EA), and PSD and shamEA treatment (PSD+ShamEA) groups (one-way ANOVA, N=12 per group; F=17.48, ***P<0.01,*). **(c)** Average frequency of EMG responses (one-way ANOVA, N=12 per group; F=10.86, ***P<0.01*). **(d)** Onset latency of EMG responses (one-way ANOVA, N=12 per group; F=6.222, ***P<0.01*). Data are presented as Mean±SEM. N indicates the number of biologically independent samples, mice per group.

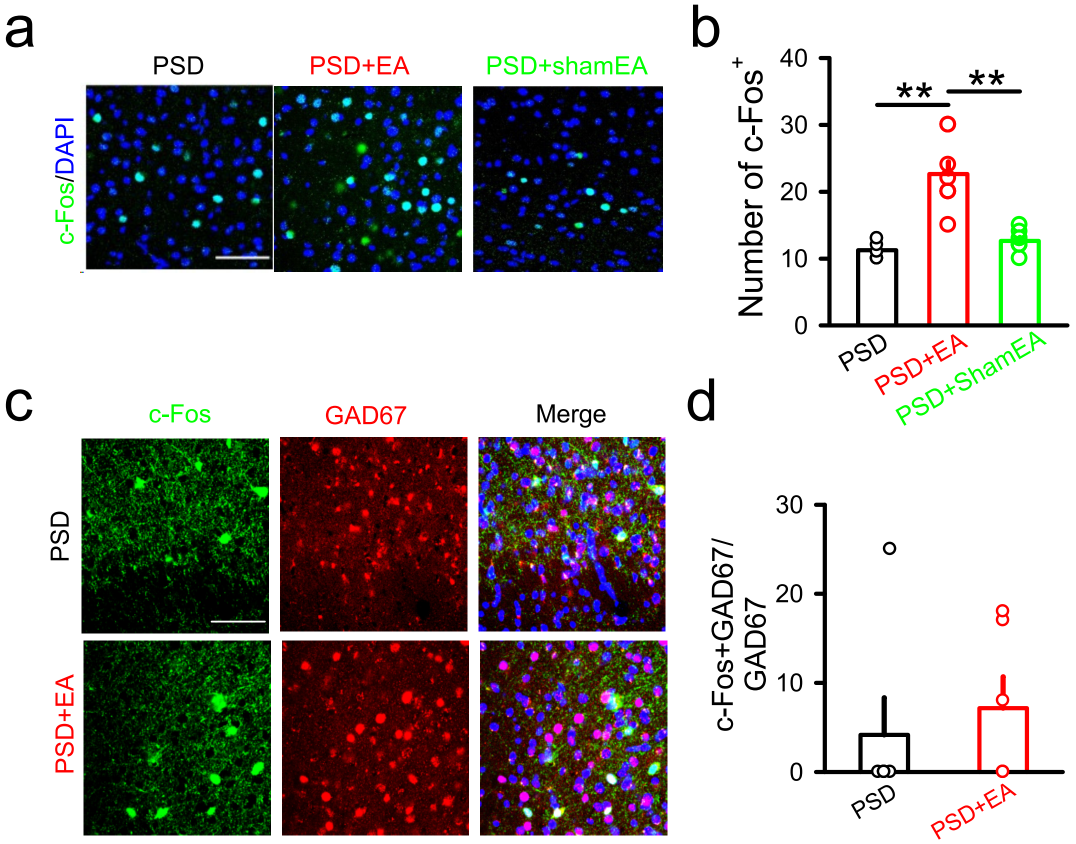

Sup Fig.3. EA-CV23-induced c-Fos expression in the contralateral M1 in PSD model mice.

(a) Representative images of c-Fos expression in the contralateral M1 after EA stimulation in the PSD, PSD+EA, and PSD+ShamEA groups. Scale bar, 50 µm. **(b)** The number of c-Fos expression in the M1 was enhanced in the EA treatment group compared to that in the PSD and PSD+ShamEA groups (one-way ANOVA, N=8 per group; F=29.81, $^{**}P<0.01$). **(c)** Representative images of c-Fos-positive and GABAergic neurons in the contralateral M1 in the PSD and PSD+EA groups. Scale bar, 50 µm. **(d)** The expression of c-Fos in GABAergic neurons was not different between the PSD and PSD+EA groups (two-tailed Student's unpaired *t* test, N=6 per group; t=0.5509, P=0.5938). Data are presented as Mean±SEM. N indicates the number of biologically independent samples, mice per group.

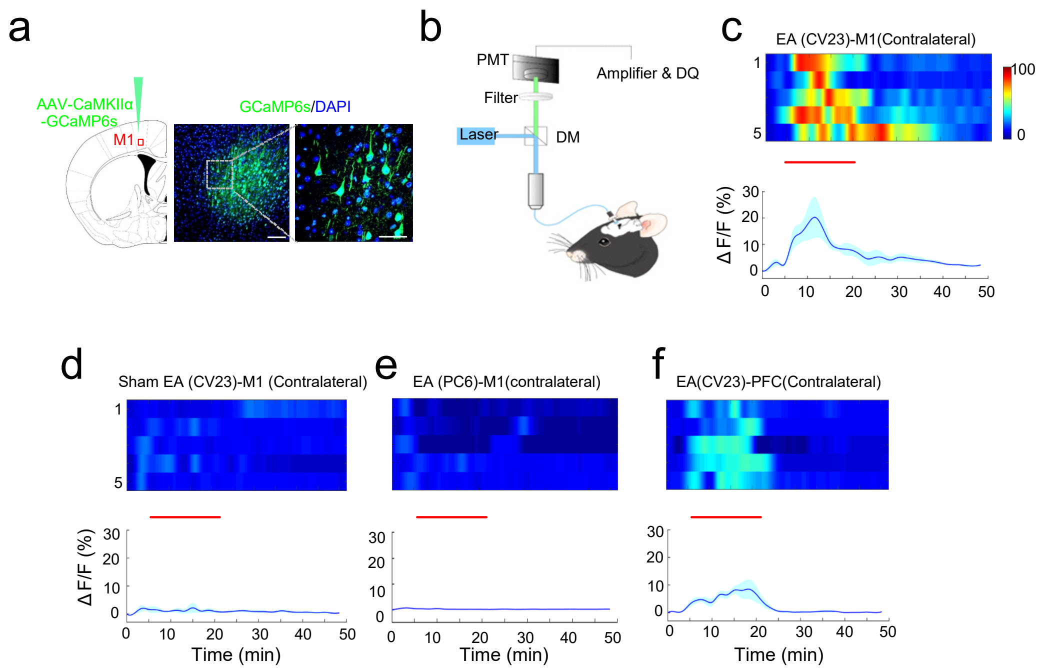

Sup Fig.4. Ca^{2+} signals in excitatory neurons recorded via fiber photometry during EA-CV23.

(a) The location of the injection site and the virus expression. Scale bar, 100 μm (left) and 50 μm (right). **(b)** Schematic diagram of fiber photometry in mice. **(c)** Heatmaps of illustrating the significant evoked Ca^{2+} signals in M1 L5 neurons in the contralateral hemisphere from PSD model mice during and after EA-CV23 (N= 5, the line indicates the mean, and the shaded area indicates the SEM). **(d-e)** Heatmaps of illustrating a few Ca^{2+} signals in M1 L5 neurons in the contralateral hemisphere of PSD model mice during and after sham EA (**d**) and EA at the PC6 acupoint (**e**). **(f)** Heatmaps of illustrating the Ca^{2+} signals in PFC neurons in the contralateral hemisphere of PSD model mice during and after EA-CV23 (N=5). Data are presented as Mean±SEM. N indicates the number of biologically independent samples, mice per group.

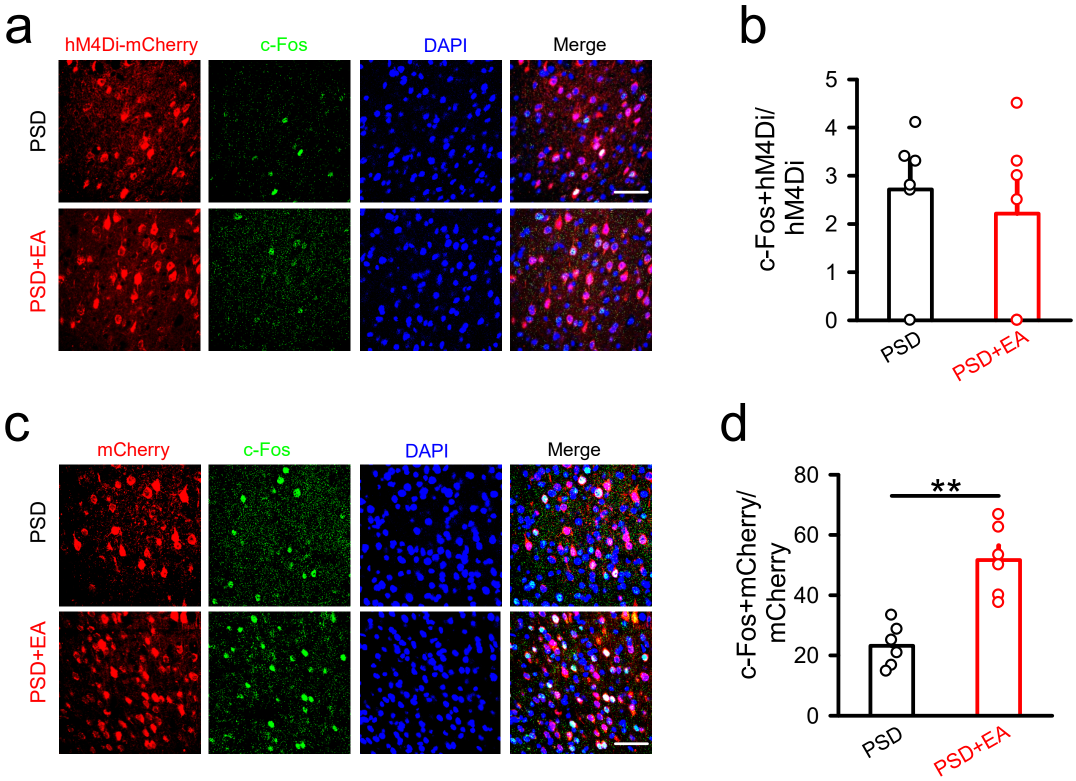

Sup Fig.5. c-Fos expression in hM4Di- or mCherry-labeled neurons with in M1.

(a) Representative images of the expression of c-Fos in AAV2/9-CaMKIIα-hM4Di-mCherry-labeled neurons in the M1 in the PSD and PSD+EA groups. Scale bar, 50 μm. **(b)** After chemogenetic inhibition of M1 neurons, the expression of c-Fos was not different between the PSD and PSD+EA groups (two-tailed Student's unpaired *t* test, N=6 per group; *t*=0.5269, *P*=0.6098). **(c)** Representative image of c-Fos expression in AAV2/9-CaMKIIα-mCherry-labeled neurons in the M1 of PSD and PSD+EA groups. Scale bar, 50 μm. **(d)** When M1 neurons were not inhibited, the expression of c-Fos was increased in the EA group compared to the PSD group (two-tailed Student's unpaired *t* test, N=6 per group; *t*=5.088, ***P*< 0.01). Data are presented as Mean±SEM. N indicates the number of biologically independent samples, mice per group.

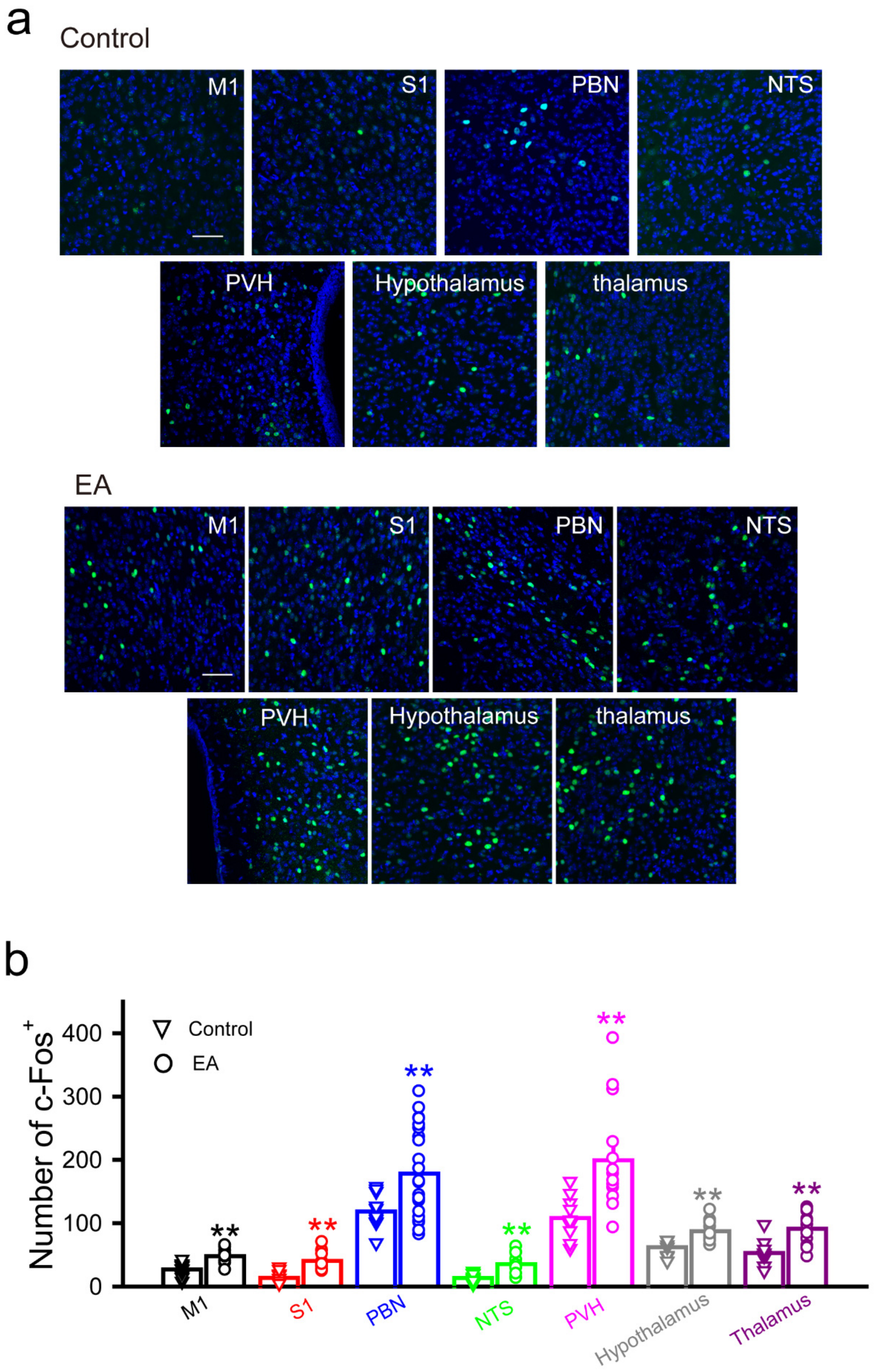

Sup Fig.6. Induction of c-Fos expression in different brain regions following EA treatment.

(a) Confocal images showing c-Fos expression in the M1, S1, PBN, NTS, PVH, hypothalamus and thalamus after control treatment or EA-CV23 stimulation. Scale bar, 50 μm. **(b)** Histogram showing c-Fos expression induced by EA-CV23 or control stimulation (two-tailed Student's unpaired t-test, n(slice)=10~20, N(mice)=3, $^{**}P< 0.01$). Data are presented as Mean ± SEM. N indicates the number of biologically independent samples, mice per group.

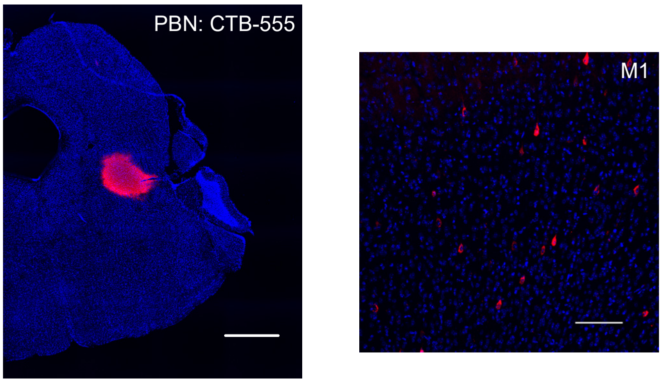

Sup Fig.7. Monosynaptic connections between the M1 and PBN.

CTB-555 was injected into the PBN (left), and labeled neurons were observed in the M1 (right). Scale bar, 1000 μm (left) and 50 μm (right)

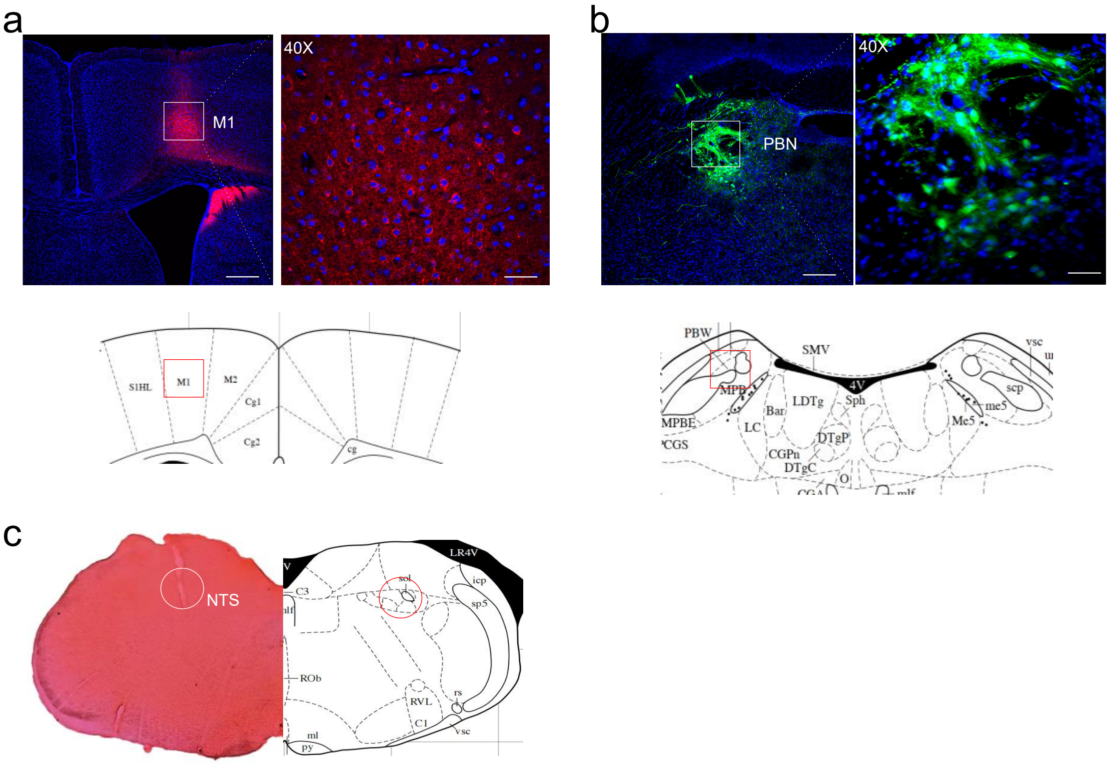

Sup Fig.8. Confirmation of virus expression and the recording site.

(a) The location of AAV2/9-CaMKIIα-ChR2-mCherry virus injection in the M1. Scale bar, 10X: 200 μm (left); 40X: 50 μm (right). **(b)** The location of AAV2/9-CaMKIIα- hM4Di -mCherry virus injection in the PBN. Scale bar, 10X: 200 μm (left); 40X: 50 μm (right). **(c)** The location of electrode implantation in the NTS.

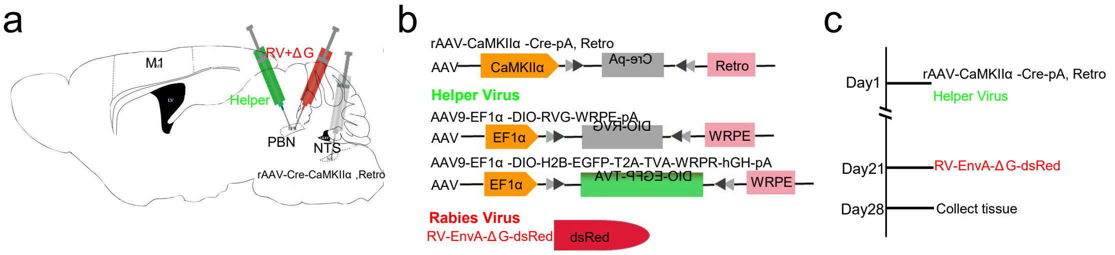

Sup Fig.9. Monosynaptic retrograde tracing with RV-ΔG.

(a) The experimental scheme for the retrograde tracing with RV. **(b)** The viruses used in the experiment included rAAV-Retro virus, helper virus, and RV virus. **(c)** Timeline of virus injection.

Supplementary Table 1 Abbreviations

**Abbreviation**	
Amyotrophic lateral sclerosis	ALS
Area under the curve	AUC
Cholera toxin subunit B	CTB
Agouti gene-related protein	AgRP
Clozapine-N-oxide	CNO
Central pattern generators	CPGs
Electroacupuncture	EA
Electromyography	EMG
Fiberoptic endoscopic evaluation of swallowing	FEES
Gamma aminobutyric acid	GABA
Green fluorescent protein	GFP
Kubota water swallowing test	WST
Layer 5	L5
Lianquan acupoint	CV23
Neuromuscular electrical stimulation	NMES
Nucleus tractus solitarii	NTS
Paraventricular hypothalamus	PVH
Parabrachial nuclei	PBN
Pharyngeal electric stimulation	PES
Post-stroke dysphagia	PSD
Prefrontal cortex	PFC
Primary motor cortex	M1
Paraformaldehyde	PFA
Neiguan	PC6
Rabies virus	RV
Rose Bengal	RB
Standardized Swallowing Assessment	SSA
Transcranial magnetic stimulation	TMS
Transcranial direct current stimulation	tDCS
Vesicular stomatitis virus	VSV
Video fluoroscopic swallowing study	VFSS

Supplementary Table 2 Experimental list

Figures	Purpose	Experiments	Findings	# of mice
**Fig. 1b-c**<br>**Sup Fig. 1b**	To detect synaptic connections between M1 neurons and the mylohyoid	(1) Injection of PRV-CMV-EGFP into the mylohyoid;<br>(2) Immunofluorescence	EGFP-positive neurons were detected in M1 L5 at 96 h after injection but not at 48 h or 72 h	5
**Sup Fig. 1d-f**	To verify the expression of the virus	(1) Injection of AAV2/9-CaMKIIα-ChR2-mCherry into the M1;<br>(2) Electrophysiological recording in *in vitro* slices	mCherry-positive labelling neurons were observed in the M1, and depolarization of the membrane potential and action potential firing were induced by blue light stimulation	3
**Fig. 1d-f**	To confirm the reliability of EMG recording for swallowing evaluation	(1) Injection of AAV2/9-CaMKIIα-ChR2-mCherry into the M1;<br>(2) EMG recording;<br>(3) Balloon pressure detection	The AUC of EMG responses in the mylohyoid was well correlated with the pharyngeal pressure	6
**Fig. 1h-k**	To determine the optimal parameters for optogenetic activation of M1 neurons to elicit EMG responses	(1) Injection of AAV2/9-CaMKIIα-ChR2-mCherry into the M1;<br>(2) EMG recording	Optogenetic stimulation parameters (8 mW, 50 Hz) for eliciting the maximum EMG responses were determined	96/8*
**Fig. 2a-c**	To establish a mouse model of ischemia	(1) Induction of photothrombosis in the M1/PFC;<br>(2) Laser speckle imaging	Blood flow was decreased in the ischemic M1 and PFC compared to the contralateral region of M1 and PFC	30/5*
**Fig. 2d-f**	To evaluate the swallowing function in ischemic mice	(1) EMG recording;<br>(2) Water consumption test	The AUC of EMG responses in the mylohyoid induced by water delivery and water consumption were decreased in mice with in M1 ischemia	50/5* in **Fig. 2d-e**<br>50/5* in **Fig. 2f**
**Fig. 2g-i**<br>**Sup Fig. 2b-d**	To test the effect of EA-CV23 in PSD model mice	(1) EMG recording;<br>(2) Water consumption test	The AUC, the amplitude, frequency, and the onset latency of EMG responses were increased by EA-CV23	48/4* in **Fig. 2g-h& Sup Fig. 2b-d**;<br>44/4* in **Fig. 2i**
**Fig. 3a-b**<br>**Sup Fig. 3**	To determine the type of neurons in the M1 activated by EA-CV23	Immunofluorescence for CaMKIIα, GAD67, and c-Fos	CaMKIIα-positive excitatory neurons in M1 L5 were activated by EA-CV23	12/2* in **Fig. 3a-b**;<br>24/3* in **Sup Fig. 3a-b**;<br>12/2* in **Sup Fig. 3c-d**
**Fig. 3c-e**	To evaluate the Ca^{2+}	(1) Injection of AAV2/9-	Individual somatic Ca^{2+} transients of	20/2*

	transients in individual neurons in the M1	CaMKIIα-GCaMP6s into the M1;<br>(2) *In vivo* transcranial two-photon imaging	excitatory neurons in M1 L5 were increased by EA-CV23 in PSD model mice	
**Sup Fig. 4**	To measure the Ca^{2+} signals in neurons in the M1/PFC	(1) Injection of AAV2/9-CaMKIIα-GCaMP6s into the M1/PFC;<br>(2) *In vivo* fiber photometry recording	The Ca^{2+} signals of excitatory neurons in M1 L5 was increased by EA-CV23 in PSD model mice	20/4* in **Sup Fig. 4c-f**
**Fig. 3f-h**<br>**Sup Fig. 5**	To explore the role of the M1 in the EA-mediated improvement of swallowing activity in PSD model mice	(1) Injection of AAV2/9-CaMKIIα-hM4Di-mCherry or AAV2/9-CaMKIIα-mCherry into the M1;<br>(2) EMG recording;<br>(3) Water consumption test;<br>(4) Immunofluorescence for c-Fos	The effects of EA-CV23 ton swallowing function and c-Fos expression was blocked by inhibition of M1 L5 excitatory neurons	48/4* in **Fig. 3f-g;**<br>44/4* in **Fig. 3h;**<br>24/4* in **Sup Fig. 5**
**Sup Fig. 6**	To assess c-Fos expression in different brain regions following EA treatment	Immunofluorescence for c-Fos	Neurons in the PBN, M1, NTS and thalamus, PVH, hypothalamus and S1, were activated by EA-CV23	6/2*
**Fig. 4a-d**	To measure c-Fos expression in the PBN following EA-CV23 in control and PSD model mice	Immunofluorescence for c-Fos	The expression of c-Fos in the PBN was increased by EA-CV23 in control and PSD model mice	12/2* in **Fig. 4a-b**;<br>12/2* in **Fig. 4c-d**
**Fig. 4e-h**	To investigate the role of the PBN in the EA-mediated improvement of swallowing activity in control and PSD model mice	(1) Injection of AAV2/9-CaMKIIα-hM4Di-mCherry into the PBN;<br>(2) EMG recording	Inhibition of PBN neurons decreased the EMG responses in the mylohyoid	60/5* in **Fig. 4e-f**<br>55/5* in **Fig. 4g-h**
**Fig. 5a-b**	To assess the neuronal activation in the PBN induced by optogenetic activation of M1 neurons	(1) Injection of AAV2/9-CaMKIIα-ChR2-mCherry into the M1;<br>(2) Immunofluorescence for c-Fos	Optogenetic activation of M1 L5 neurons increased the expression of c-Fos expression in the PBN	12/2*
**Fig. 5c-d**	To explore the role of PBN neurons in swallowing activity induced by activation of M1 neurons	(1) Injection of AAV2/9-CaMKIIα-ChR2-mCherry into the M1;<br>(2) Injection of AAV2/9-CaMKIIα-hM4Di-EGFP into the PBN;	Inhibition of PBN neurons decreased the mylohyoid activity induced by optogenetic activation of M1 neurons	12

		(3) EMG recording		
**Fig. 5e-f**	To directly investigate the role of the M1-PBN circuit in swallowing function	(1) Injection of AAV2/9-CaMKIIα-ChR2-mCherry into the M1;<br>(2) Fiber implantation in the PBN;<br>(3) EMG recording	Selective activation of the M1-PBN neural circuit increased EMG responses in the mylohyoid	16
**Fig. 5g**	To verify the existence of synaptic connections between the M1 and PBN using the anterograde transsynaptic tracing with AAV	(1) Injection of AAV2/1-hSyn-Cre into the M1, and injection of AAV2/9-CAG-Dio-EGFP into the PBN;<br>(2) Immunofluorescence	GFP-labeled neurons were observed in the PBN, suggesting the existence of monosynaptic connections between the M1 and PBN	5
**Fig. 5h**<br>**Sup Fig. 7**	To verify the existence of synaptic connections between the M1 and PBN using an anterograde vesicular stomatitis virus or CTB-555	(1) Injection of VSV-mCherry into the M1;<br>(2) Injection of CTB-555 into the PBN<br>(3) Immunofluorescence	mCherry-labeled neurons were observed in the PBN (**Fig. 5h**), and labeled neuron in the M1 (**Sup Fig. 7**) suggesting the existence of synaptic connections between the M1 and PBN	5 in **Fig. 5h**<br>3 in **Sup Fig. 7**
**Fig. 6a-c**	To determine the role of the NTS in swallowing activity and the effect of EA on PSD	(1) Injection of AAV2/9-CaMKIIα-hM4Di-mCherry into the NTS;<br>(2) EMG recording;<br>(3) Immunofluorescence for c-Fos	Inhibition the NTS neurons decreased swallowing activity, and the expression of c-Fos in the NTS was enhanced by EA-CV23 treatment in PSD model mice.	10/2* in **Fig. 6a**;<br>16/2* in **Fig. 6b-c**
**Fig. 6d**	To explore the synaptic connections between the PBN and NTS	(1) Injection of AAV2/1-hSyn-Cre virus into the PBN, and injection of AAV2/9-CAG-Dio-EGFP into the NTS<br>(2) Immunofluorescence	EGFP-labeled neurons were observed in the NTS.	5
**Fig. 6e-f**<br>**Sup Fig. 8**	To explore whether the neuronal activity in the NTS could be modulated by the M1 and PBN	(1) Injection of AAV2/9-CaMKIIα-ChR2-mCherry into the M1, and injection of AAV2/9-CaMKIIα-hM4Di-mCherry or AAV2/9-CaMKIIα-mCherry into the PBN;<br>(2) *In vivo* electrophysiological recording;<br>(3) Immunofluorescence	The neuronal spikes in the NTS discharged higher after M1 neurons activation, whereas inhibition of PBN neurons prevented the M1 neurons activation-induced enhancement of neuronal spikes in the NTS.	32/4*
**Fig. 6g-h**	To determine whether the M1, PBN and NTS together regulate the	(1) Injection of AAV2/9-CaMKIIα-ChR2-mCherry into the M1, and injection of	Inhibition/activation of PBN and NTS neurons decreased/increased M1 neurons activation-induced EMG	24/4*in **Fig. 6g**;<br>36/6* in **Fig. 6h**

	swallowing function and the effect of EA-CV23	AAV2/9-CaMKIIα-hM4Di/hM3Dq-mCherry into the PBN and NTS;<br>(2) EMG recording	responses and prevented the EA-CV23-mediated increase in EMG responses.	
**Fig. 6i**<br>**Sup Fig. 9**	To explore whether there are synaptic connections among the M1, PBN, NTS neural circuit	(1) Injection of rAAV-CaMKIIα-Cre-Retro into the NTS and injection of RV-ΔG, AAV2/9-Dio-TVA and AAV2/9-Dio-RVG into the PBN<br>(2) Immunofluorescence	Starter neurons (i.e., PBN neurons projecting to NTS) were observed in the PBN and few fluorescence-labeled neurons were observed in the M1.	5
**Fig. 6j-k**	To assess neural activity in the NTS after inhibition of PBN neurons innervated by the M1	(1) Injection of AAV2/1-CaMKIIα-Cre into the M1 and injection of AAV2/9-hSyn-Dio-hM4Di into the PBN<br>(2) *in vivo* electrophysiology	Selectively inhibition of PBN neurons innervated by the M1 could decrease the neuronal activity in the NTS.	5
**Fig. 6l**	To investigate the role of the direct M1-PBN-NTS neural circuit in regulating swallowing function	(1) Injection of AAV2/1-CaMKIIα-Cre virus into the M1, and injection of AAV2/9-EF1α-Dio-ChR2-EYFP into the PBN, and fiber implanted into NTS;<br>(2) *In vivo* EMG recording	Selectively activation of the terminals of M1-innervated PBN neurons in the NTS increased EMG responses, but this activity was lower than that induced by M1 neurons activation.	12/2*
	Total=879<br>N (Normal)=436<br>N (Normal+EA)=45<br>N (Stroke)=201<br>N (Stroke+EA) =197			

Note：* The total number of mice/ the total number of groups in the experiment.
