# Supplementary material for: Electroacupuncture improves swallowing function in a post-stroke dysphagia mouse model by activating the motor cortex inputs to the nucleus tractus solitarii through the parabrachial nuclei
Source: Nat Commun. 2023 Feb 13;14:810. doi: 10.1038/s41467-023-36448-6 (PMC9925820; doi:10.1038/s41467-023-36448-6)
Supplement: Supplementary file 3 — Reporting Summary [file 41467_2023_36448_MOESM3_ESM.pdf]

nature portfolio

Corresponding author(s): Nenggui Xu
Last updated by author(s): Nov 25, 2022

Reporting Summary

Nature Portfolio wishes to improve the reproducibility of the work that we publish. This form provides structure for consistency and transparency in reporting. For further information on Nature Portfolio policies, see our Editorial Policies and the Editorial Policy Checklist.

Statistics

For all statistical analyses, confirm that the following items are present in the figure legend, table legend, main text, or Methods section.

n/a	Confirmed	
☐	☒	The exact sample size (n) for each experimental group/condition, given as a discrete number and unit of measurement
☐	☒	A statement on whether measurements were taken from distinct samples or whether the same sample was measured repeatedly
☐	☒	The statistical test(s) used AND whether they are one- or two-sided<br>*Only common tests should be described solely by name; describe more complex techniques in the Methods section.*
☐	☒	A description of all covariates tested
☐	☒	A description of any assumptions or corrections, such as tests of normality and adjustment for multiple comparisons
☐	☒	A full description of the statistical parameters including central tendency (e.g. means) or other basic estimates (e.g. regression coefficient) AND variation (e.g. standard deviation) or associated estimates of uncertainty (e.g. confidence intervals)
☐	☒	For null hypothesis testing, the test statistic (e.g. F, t, r) with confidence intervals, effect sizes, degrees of freedom and P value noted<br>*Give P values as exact values whenever suitable.*
☒	☐	For Bayesian analysis, information on the choice of priors and Markov chain Monte Carlo settings
☒	☐	For hierarchical and complex designs, identification of the appropriate level for tests and full reporting of outcomes
☒	☐	Estimates of effect sizes (e.g. Cohen's d, Pearson's r), indicating how they were calculated

Our web collection on statistics for biologists contains articles on many of the points above.

Software and code

Policy information about availability of computer code

Data collection	Nikon A1 ; Plexon(NeuroExplorer 5.022; Offline Sorter v4) ; Spike2(8.09) ; LabChar (7.2) ; NIS-Elements Viewer (4.5)
Data analysis	SPSS 21 ; GraphPad Prism 8 ; ImageJ 1.63q

For manuscripts utilizing custom algorithms or software that are central to the research but not yet described in published literature, software must be made available to editors and reviewers. We strongly encourage code deposition in a community repository (e.g. GitHub). See the Nature Portfolio guidelines for submitting code & software for further information.

Data

Policy information about availability of data

All manuscripts must include a data availability statement. This statement should provide the following information, where applicable:
- Accession codes, unique identifiers, or web links for publicly available datasets
- A description of any restrictions on data availability
- For clinical datasets or third party data, please ensure that the statement adheres to our policy

All data supporting the findings of this study are included in the article and its supplementary information files and from the corresponding author. Source data are provided with this paper.

Field-specific reporting

Please select the one below that is the best fit for your research. If you are not sure, read the appropriate sections before making your selection.

- [x] Life sciences
- [ ] Behavioural & social sciences
- [ ] Ecological, evolutionary & environmental sciences

For a reference copy of the document with all sections, see nature.com/documents/nr-reporting-summary-flat.pdf

Life sciences study design

All studies must disclose on these points even when the disclosure is negative.

Sample size	The sample size for mice used in the study was referred to the previous studied published in Nature or Nature Neuroscience. Reference：（PMID: 34646018 or PMID: 34992295）
Data exclusions	The data was excluded if the virus location was incorrect and the exclusion criteria was presented in the article.
Replication	Each experiment in the study was replicated more than 3 times, and most of these experiments were performed at least 5 times.
Randomization	Samples were randomly grouped by random number table.
Blinding	Sample grouping, experiment operators and data analysts are blinded to each other.

Reporting for specific materials, systems and methods

We require information from authors about some types of materials, experimental systems and methods used in many studies. Here, indicate whether each material, system or method listed is relevant to your study. If you are not sure if a list item applies to your research, read the appropriate section before selecting a response.

Materials & experimental systems

n/a	Involved in the study	
[ ]	[x]	Antibodies
[x]	[ ]	Eukaryotic cell lines
[x]	[ ]	Palaeontology and archaeology
[ ]	[x]	Animals and other organisms
[x]	[ ]	Human research participants
[x]	[ ]	Clinical data
[x]	[ ]	Dual use research of concern

Methods

n/a	Involved in the study	
[x]	[ ]	ChIP-seq
[x]	[ ]	Flow cytometry
[x]	[ ]	MRI-based neuroimaging

Antibodies

Antibodies used	rabbit anti c-Fos (Cell Signaling Technology, 2250s); Alexa Fluor 488-conjuated goat-anti-rabbit (ThermoFisher, A11034; Alexa Fluor 594-conjuated donkey anti-rabbit (ThermoFisher, A21207)
Validation	https://www.cellsignal.com/products/primary-antibodies/c-fos-9f6-rabbit-mab/2250; https://www.thermofisher.cn/cn/zh/antibody/product/Goat-anti-Rabbit-IgG-H-L-Highly-Cross-Adsorbed-Secondary-Antibody-Polyclonal/A-11034; https://www.thermofisher.cn/cn/zh/antibody/product/Donkey-anti-Rabbit-IgG-H-L-Highly-Cross-Adsorbed-Secondary-Antibody-Polyclonal/A-21207;

Animals and other organisms

Policy information about studies involving animals; ARRIVE guidelines recommended for reporting animal research

Laboratory animals	C57BL/6 male mice with 2~3 months old were purchased from Guangzhou University of Chinese Medicine
Wild animals	not used
Field-collected samples	no field-collected samples
Ethics oversight	All surgery and experimental procedures in this study were performed in accordance with the guidelines of Guangzhou University of Chinese Medicine Committee for Care and Use of Research Animals

Note that full information on the approval of the study protocol must also be provided in the manuscript.
